# Dataset for homologous proteins in *Drosophila melanogaster* for SARS-CoV-2/human interactome

**DOI:** 10.1016/j.dib.2020.106082

**Published:** 2020-07-26

**Authors:** Mushtaq Hussain, Nusrat Jabeen, Sanya Shabbir, Nasir Udin, Basma Aziz, Anusha Amanullah, Fozia Raza, Ayesha Ashraf Baig

**Affiliations:** a Bioinformatics and Molecular Medicine Research Group, Dow Research Institute of Bitoechnology and Biomedical Sciences, Dow College of Biotechnology, Dow University of Health Sciences, Karachi, Pakistan; b Department of Microbiology, University of Karachi, Karachi, Pakistan; c Faculty of Computer Science, IBA, Karachi, Pakistan

**Keywords:** SARS-CoV-2, *Drosophila melanogaster*, COVID-19, Animal modelling, Interactome

## Abstract

Animal modelling for infectious diseases is critical to understand the biology of the pathogens including viruses and to develop therapeutic strategies against it. Herein, we present the sequence homology and expression data analysis of proteins found in *Drosophila melanogaster* that are orthologous to human proteins, reported as components of SARS-CoV-2/Human interactome. The dataset enlists sequence homology, query coverage, domain conservation, OrthoMCL and Ensembl Genome Browser support of 326 proteins in *D.melanogaster* that are potentially orthologous to 417 human proteins reported for their direct physical interactions with 28 proteins encoded by SARS-CoV-2 genome. Expression of these *D.melanogaster* orthologous genes in 26 anatomical positions are also plotted as heat maps in 27 sets, corresponding to the potential protein interactors for each viral protein. The data could be used to direct experiments and potentially predict their phenotypic and molecular outcome in order to dissect the biological roles and molecular functionality of SARS-CoV-2 proteins in a convenient animal model system like *D.melanogaster*.

Specifications Table

**Table:** 

**Subject**	Biochemistry, Genetics and Molecular Biology (General)
**Specific subject area**	Bioinformatics, Animal Modelling
**Type of data**	Image Figure Excel Sheets
**How data were acquired**	Cytoscape v3.7.1 ClustVis (https://biit.cs.ut.ee/clustvis/) Ensembl Genome Browser (https://asia.ensembl.org/index.html) Fly Atlas (http://flyatlas.org/atlas.cgi) NCBI BLAST (https://blast.ncbi.nlm.nih.gov/Blast.cgi) OrthoMCL (https://orthomcl.org/orthomcl/) UniProt (https://www.uniprot.org/)
**Data format**	Raw analysed Filtered
**Parameters for data collection**	Network Data and Heat maps for expression were generated using Cytoscape and ClustVis, respectively, assessed using Intel(R) Xeon (R) CPU X5660 GHz, 2.79 GHz GPU, 32GB RAM.
**Description of data collection**	The orthologous genes were identified using UniProt, NCBI Blast, OrthoMCL and Ensembl Genome Browser. Expression data of the orthologous genes in *D.melanogaster* were collected from Fly Atlas.
**Data source location**	Institution: Bioinformatics and Molecular Medicine Research Group, Dow Research Institute of Biotechnology and Biomedical Sciences, Dow College of Biotechnology, Dow University of Health Sciences City: Karachi Country: Pakistan
**Data accessibility**	Repository name: Mendeley Data identification number: 10.17632/h2dmwzk4z3.2 Direct URL to data: https://data.mendeley.com/datasets/h2dmwzk4z3/2

**Value of the Data**

• The data enlist proteins in D.melanogaster that are homologous to the human proteins interactors with SARS-CoV-2 proteins, therefore the data is useful in accessing the suitability of D.melanogaster as a model organism to study the biology of SARS-CoV-2 genes.
• Virologists, fly biologists, pharmacologists and protein biochemists could be benefited from the present dataset to investigate the partner protein interactions between the host and SARS-CoV-2 and their molecular consequences.
• The dataset could be exploited to screen inhibitors and/or disruptors designed against SARS-CoV-2 for their efficacy and safety in model system like D.melanogaster.
• The dataset could also be used to identify target genes for exploring expressional changes in D.melanogaster following heterologous cloning and expression of SARS-CoV-2 proteins and upon exposure of different drugs employed in the management of COVID-19.

## 1 Data description

**Fig. 1:**
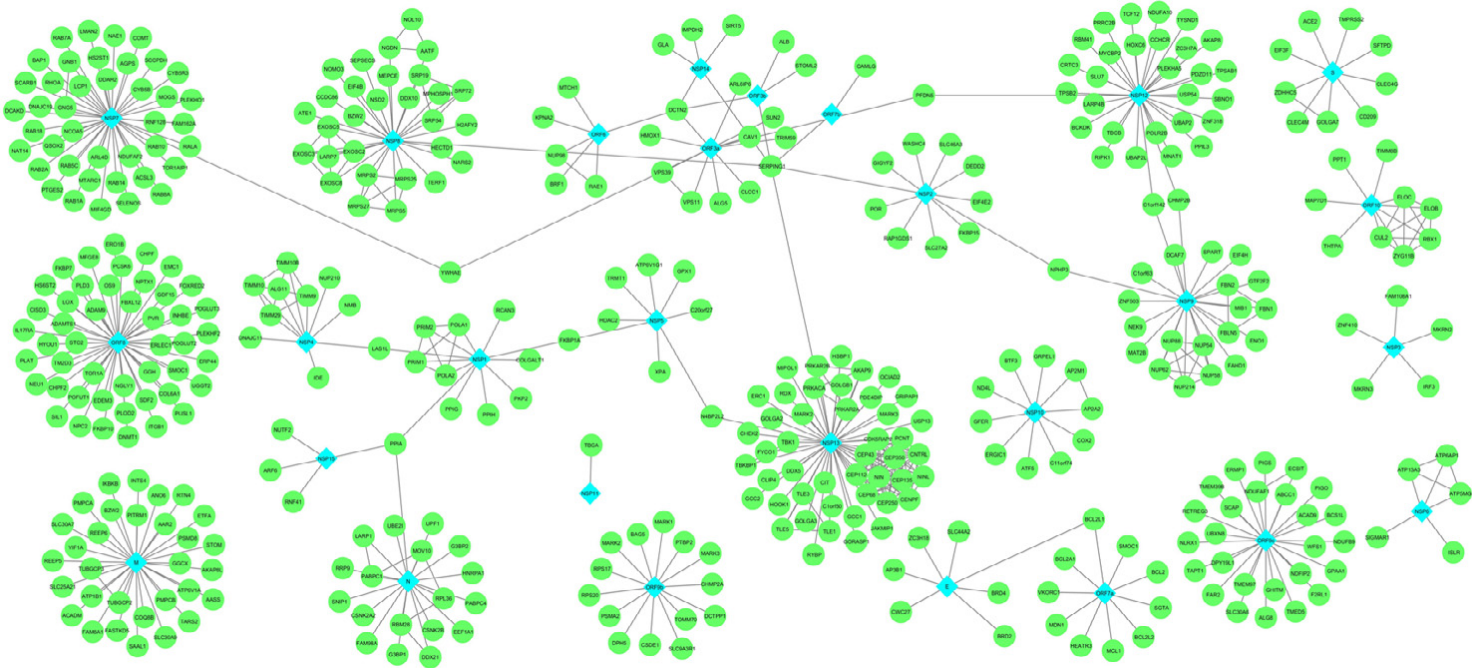
Simplified molecular interaction network between SARS-CoV-2 proteins (blue diamonds) and human proteins (green circle).

The dataset is composed of both raw and analysed data, organized in three directories: Interactome, Orthologues and Expression, made available at https://data.mendeley.com/datasets/h2dmwzk4z3/2 and supplementary files. Interactome directory contains simplified network file (cytoscape format) of SARS-CoV-2/human Interactome, coalescing two previous studies [1,2]. Jpeg image of the network is also present in the same directory. The combined network shows 449 nodes and 582 edges (interactions) as shown in Fig. 1.

**Fig. 2:**
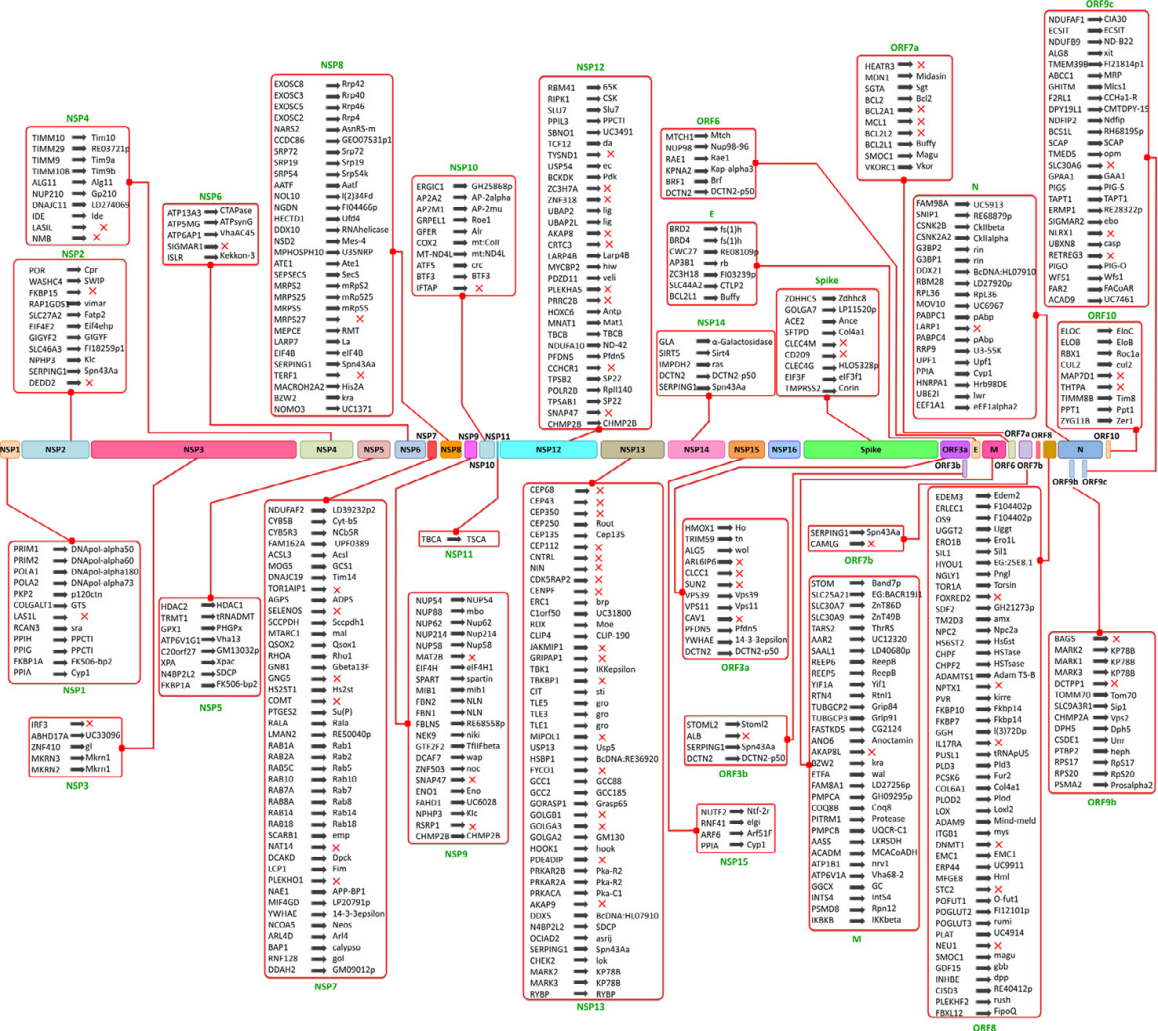
Schematic and scaled representation of SARS-CoV-2 genome with human protein interactors are indicated in the blocks linking respective viral proteins. Presence or absence of *D.melanogaster* orthologues of human proteins are mentioned by name or red cross, respectively.

Orthologues directory stores an excel file where all human partner protein interactors corresponding to the 28 viral proteins are listed. Accession number of the proteins and domains are also indicated at the corresponding positions. Name of the potential *D.melanogaster* orthologues with accession numbers, sequence identity, query coverage, domains and Ensembl Genome Browser and OrthoMCL support (accession numbers) are also tabulated at the respective rows. In case, the human partner protein was proposed as drug target in a previous study [1] it has also been indicated. An explanatory README file is also placed in the orthologues directory. The final list of the human interactors of the SARS-CoV-2 viral proteins along with the identified orthologues in *D.melanogaster* is schematically represented in Fig. 2. For clarity the enlarged image of the same is also placed in interactome directory.

**Fig. 3:**
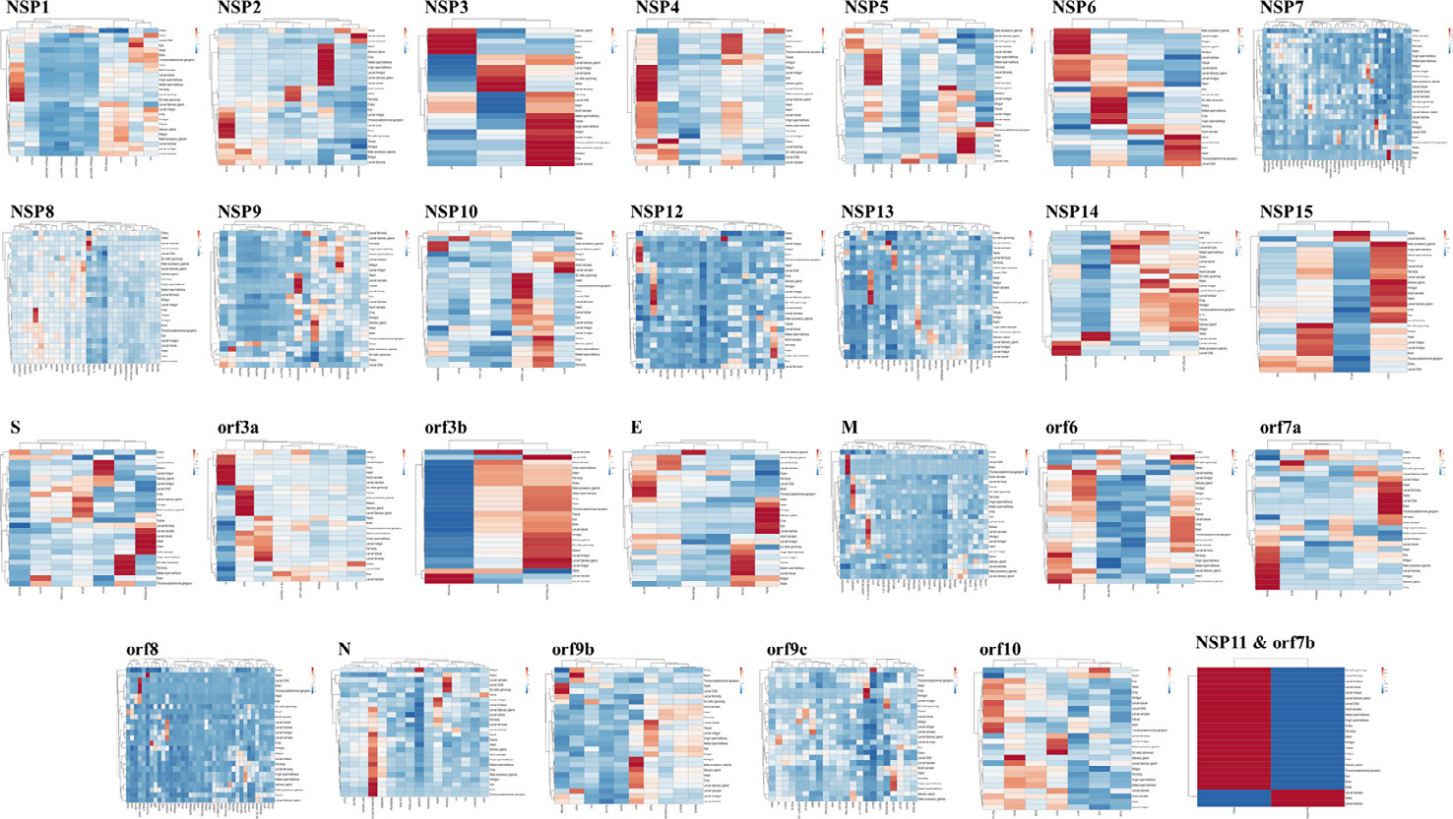
Heat maps showing the expression of *D.melanogaster* genes, orthologous to human proteins interactors with SARS-CoV-2 proteins as labelled on the top of each map.

Expression directory stores an excel file where gene expression data retrieved from Fly Atlas [3] of the identified orthologous genes in *D.melanogaster* for 26 different anatomical positions are tabulated. The expression values are the representation of the mRNA enrichment of the gene of interest at respective anatomical sites. The probe selected for the enrichment is also indicated in the excel file. The heat maps of these gene sets, developed using ClustVis [4], corresponding to the potential interacting SARS-CoV-2 proteins are shown in Fig. 3 with scales as indicators of expression. For better resolution, pdf files of each expression map are also stored in the expression directory.

## 2 Experimental design, materials and methods

Simplified SARS-CoV-2/human interactome was constructed in Cytoscape v3.7.1 by first creating manual nodes and edges of 332 human proteins that have shown to bind with the 26 SARS-CoV-2 proteins in the bait experiment, designed for repurposing drugs against SARS-CoV-2 [1]. Additional 88 human genes and 2 viral proteins were picked from another study [2], conducted to identify the dysregulation of human genes during the viral infection. Both networks were combined using union sub-function to remove redundant repetition of the genes (Fig. 1). Given the objective of the dataset and to maintain clarity, interactions between human proteins within the network were removed.

Primary sequence of all human proteins were retrieved from UniProt and subjected to genome specific BLAST for the identification of orthologues in *D.melanogaster*. Orthologous proteins were identified on the basis of query coverage, sequence identity and domain conservation. Further support of orthology was gathered from *D.melanogaster* genome assembly at Ensembl genome browser [5] and OrthoMCL [6]. In *D.melanogaster* genome specific BLAST, fruit fly protein sequences which share equal or more than 20% sequence identity, covers equal or more than 15% of target (human) sequence and contain same functional domain(s) were considered as positive hit for the homology of respective human gene. In Ensembl Genome Browser, if the orthologous gene of *D.melanogaster* is listed in the respective human gene page, it was accounted as a positive identification. In OrthoMCL, homologous protein in *D.melanogaster* were also identified by BLAST search of the human protein sequence at E-value thereshold less than 1 × 10^−5^. Operationally, the final decision for the presence of orthologue in *D.melanogaster* is based on the principle when at least both NCBI genome specific BLAST and OrthoMCL identify same protein (indicated by the annotation number) as an orthologous protein in *D.melanogaster* for the target human protein.

Expression values of the identified orthologues of *D. melanogaster* were retrieved from Fly Atlas [3] for 26 anatomical positions using specific probes. The values then tabulated in the MS Excel and converted in Text (Tab delimited) format. The values then used to developed heat maps using ClustVis [4] where anatomical positions (rows) and expression values of genes (columns) were clustered on the basis of strongest correlation and arranged as tightest cluster first.

## 3 Ethics statement

The dataset is based on bioinformatic analysis, therefore, no animal has been used and/or harmed in the present investigation.

## Declaration of Competing Interest

The authors declare that they have no known competing financial interests or personal relationships which have, or could be perceived to have, influenced the work reported in this article.

## Appendix Supplementary materials

Image, application 1

Image, application 1

Image, application 1

Image, application 1
